# Multiple sclerosis genetic and non-genetic factors interact through the transient transcriptome

**DOI:** 10.1038/s41598-022-11444-w

**Published:** 2022-05-09

**Authors:** Renato Umeton, Gianmarco Bellucci, Rachele Bigi, Silvia Romano, Maria Chiara Buscarinu, Roberta Reniè, Virginia Rinaldi, Raffaella Pizzolato Umeton, Emanuele Morena, Carmela Romano, Rosella Mechelli, Marco Salvetti, Giovanni Ristori

**Affiliations:** 1 Department of Informatics and Analytics, Dana-Farber Cancer Institute, Boston, MA USA; 2 Department of Biological Engineering, Department of Mechanical Engineering, Massachusetts Institute of Technology, Cambridge, MA USA; 3 Department of Biostatistics, Harvard T.H. Chan School of Public Health, Boston, MA USA; 4 Department of Pathology and Laboratory Medicine, Weill Cornell Medicine, New York, NY USA; 5 Department of Neurosciences, Mental Health and Sensory Organs, Centre for Experimental Neurological Therapies (CENTERS), Sapienza University of Rome, Rome, Italy; 6 Neuroimmunology Unit, IRCCS Fondazione Santa Lucia, Rome, Italy; 7 Department of Neurology, UMass Memorial Health Care, Worcester, MA USA; 8 University of Massachusetts Medical School, Worcester, MA USA; 9 Department of Neurology, Massachusetts General Hospital, Boston, MA USA; 10 Harvard Medical School, Boston, MA USA; 11 IRCCS San Raffaele Pisana, Rome, Italy; 12 San Raffaele Roma Open University, Rome, Italy; 13 IRCCS Istituto Neurologico Mediterraneo Neuromed, Pozzilli, Italy

**Keywords:** Immunopathogenesis, Multiple sclerosis, Functional genomics, Gene regulation, Genomics, Genome informatics, Immunology, Neurology, Pathogenesis

## Abstract

A clinically actionable understanding of multiple sclerosis (MS) etiology goes through GWAS interpretation, prompting research on new gene regulatory models. Our previous investigations suggested heterogeneity in etiology components and stochasticity in the interaction between genetic and non-genetic factors. To find a unifying model for this evidence, we focused on the recently mapped transient transcriptome (TT), that is mostly coded by intergenic and intronic regions, with half-life of minutes. Through a colocalization analysis, here we demonstrate that genomic regions coding for the TT are significantly enriched for MS-associated GWAS variants and DNA binding sites for molecular transducers mediating putative, non-genetic, determinants of MS (vitamin D deficiency, Epstein Barr virus latent infection, B cell dysfunction), indicating TT-coding regions as MS etiopathogenetic hotspots. Future research comparing cell-specific transient and stable transcriptomes may clarify the interplay between genetic variability and non-genetic factors causing MS. To this purpose, our colocalization analysis provides a freely available data resource at www.mscoloc.com.

## Introduction

A large body of literature agrees that regulatory genomic intervals, especially those encompassing enhancers, are enriched with disease-associated DNA elements. Most of this evidence comes from genome wide association studies (GWAS) based on single polymorphism nucleotides (SNPs) representing common variants^1–5^, even though a recent study showed that low-frequency and rare coding variants may somewhat contribute to multifactorial diseases^6^. Several characteristics of regulatory disease-associated genetic variants complicate GWAS interpretation, prompting research on new gene regulatory models: (i) SNPs are chosen as haplotypes to spare the genotyping work needed for the large number of samples used in GWAS, therefore fine mapping and epigenetic studies are required to integrate GWAS data^7–10^; (ii) a fraction of supposedly causal disease-associated variants directly alters recognizable transcription factor binding motifs as it might be expected, according to their regulatory function^4^; (iii) the identified GWAS signals are likely to exert highly contextual (i.e., time- and position-dependent) regulatory effects, that may change according to the tissue and to the time when they receive an input from inside or outside the cell. In summary, current gene regulatory models help only in part to fully detail which disease-associated SNP signals are causal, and by which exact mechanisms they are causal. Recent studies on the biological spectrum of human DNase I hypersensitive sites (DHSs), that are disease-associated markers of regulatory DNA, may help to better rework GWAS data and particularly to contextualize the genomic variants according to tissue/cell states and to gene body colocalization of DHSs^11^. In this context, the latest version of the Genotype-Tissue Expression project may provide further insights into the tissue specificity of genetic effects, supporting the link between regulatory mechanisms and traits or complex diseases^12^.

Such layers of complexity are in agreement with our previous investigations on the interplay between genetic and non-genetic factors contributing to multiple sclerosis (MS) development: twin pairs studies^13,14^; disease modelling suggested stochastic phenomena (i.e., random events not necessarily resulting in disease in all individuals) contributing to the disease onset and progression^15,16^; bioinformatics analyses determined a significant enrichment of binding motifs for Epstein-Barr virus (EBV) nuclear antigen 2 (EBNA2) and vitamin D receptor (VDR) in genomic regions containing MS-associated GWAS variants^17^. We also demonstrated that genomic variants of *EBNA2* resulted to be MS-associated^18^, and other groups expanded our findings showing that enrichment of EBNA2-binding regions on GWAS DNA intervals is involved in the pathogenesis of autoimmune disorders, including MS^19^. The role of EBV proteins and/or VDR as key transcriptional regulators in MS falls within well-known sero-epidemiological evidences on the virus as risk factor for disease development^20^, and on the vitamin deficiency associated to different disease prevalence in diverse geographic areas^21^. Recent works have even reinforced the EBV causal role and its mechanistic link for MS development^22,23^.

A recent sequencing innovation (namely, TT-seq) allowed to map the transient transcriptome that has a typical half-life within minutes, compared to stable RNA elements, such as protein-coding mRNAs, long-noncoding RNAs, and micro-RNAs, that persists at least a few hours^24–26^. The transient transcriptome (TT) includes mostly enhancer RNAs (eRNA), short intergenic non-coding RNAs (sincRNA) and antisense RNAs (asRNA). eRNAs are bidirectionally transcribed by mammalian genome regions having specific histone modifications to finely regulate chromatin conformation and transcription. Unlike promoters, enhancers can execute their functions regardless of orientation, position and spatial segregation from target genes that may be affected both in cis and in trans by eRNAs. Most sincRNAs are defined as genomic regions located within 10 kbp of a GENOME mRNA transcription start site. Overall, these transient RNAs (trRNA) are relatively short in length, generally lack a secondary structure, and would not present those chemical modifications that characterize unidirectional and polyadenylated stable RNAs^24,27^. Other recent works based on time-resolved analysis, agree on the eRNAs very rapid functional dynamics model while interacting with the transcriptional co-activator acetyltransferase CBP/p300 complex^28,29^. This confirms the highly contextual role of eRNAs through the control of transcription burst frequencies, which are known to influence cell-type-specific gene expression profiles^30^. Along these lines, a recent study showed that T cells selectively filter oscillatory signals within the minute timescale^31^, further supporting the aforementioned model.

On these bases, we leveraged the recent sequencing innovations in the mapping of the transient transcriptome, in particular the work by Michel et al. on T lymphocytes (that are known to play a major role in MS pathogenesis), that applied both the TT-seq and the RNA-seq protocol in Jukart cells during their immediate response to the stimulation with ionomycin and phorbol 12-myristate 13-acetate (PMA). Michel's study allowed to compare the trRNAs and mRNAs with high temporal resolution, showing that TT-seq, but not RNAseq, caught rapid changes in transcriptional activity just after 15 min after stimulation^24,25^. We hypothesized that MS-associated GWAS signals prevalently fall within regulatory regions of DNA coding for trRNAs. In theory, the genomic intervals coding for this transient transcriptome may be the hotspots where temporospatial occurrences may coalesce and so contribute to physiological (developmental and/or adaptive) outcomes, or possibly give rise to disease onset or progression. This study is aimed at verifying this working hypothesis through a colocalization analysis and its further dissection in the context of MS.

## Results

### MS-associated GWAS signals colocalize with regulatory regions of DNA plausibly coding for trRNAs

We set up our region-of-interest (ROI) inside GWAS catalogue^32^ by considering all MS GWAS that were published, extracting all SNP positions, and creating a single set of genomic coordinates that therefore encompass all GWAS-derived or GWAS-verified signal for MS. We then refined the SNP list by pruning out about 1.5% of the SNPs as they did not contain intelligible genomic annotations or were duplicates. The final ROI list is reported in Additional File: Table S1 and consists of 603 unique single-nucleotide regions; to provide a “threshold” against which the match ROI <  > Database would be benchmarked, we used 107,423 regions as Universe, that corresponded to the signals coming from the entire GWAS Catalog.

Next, we matched through colocalization analyses our ROI with lists of regions resulting from the work by Michel et al., which mapped the transient and stable transcriptome captured by TT-seq after T cell stimulation^24^. We found a significant enrichment of MS-associated genetic variants in the transient transcriptome (*p*-value = 2.80 × 10^−9^; Table 1). Of note, when we split the transcriptome list in two subsets for long (≥ 60 min) and short (< 60 min) half-life, we found that only the short half-life subset significantly colocalized with the ROI (*p*-value 2.06 × 10^−8^ vs. 0.09). This finding was indicative of the relationship between MS-associated GWAS signals and the regulatory regions of DNA coding for trRNAs.

**Table 1:** Enrichment of MS-associated genetic variants in lists of T-cell transient transcripts extracted from Michel et al., 2017.

List	− log (*p*-value)	*p*-value	Odds ratio	Support	List size
Whole transient transcriptome	8.55	**2.80 × 10**^**−9**^	1.65	241	22,126
Short half-life transcripts	7.68	**2.06 × 10**^**−8**^	1.63	209	20,143
Long half-life transcripts	1.05	0.09	1.29	35	1993

The whole transcriptome list was split in two sub-lists depending on the transcripts’ half-life: short (< 60′) and long (≥ 60′), respectively. Results are considered significant at *p* < 0.05 and are highlighted in bold.

When we further dissected the mapping of the ROI colocalization signals, we found a significant excess of intergenic and intron regions (as anticipated), as well as their prevalent distribution away from the transcription start site (TSS; Figure Supplement 1A). Notably, when we extended this analysis to GWAS data coming from other multifactorial diseases or traits, dividing immune-mediated and other complex conditions, we found highly comparable profiles (supplementary Fig. S1B–C, Additional File: Table S2), suggesting that the colocalization between MS-associated DNA intervals and intergenic or intronic sequences, plausibly referring to trRNA coding regions, is shared by the genetic architecture of most multifactorial disorders.

**Figure 1:**
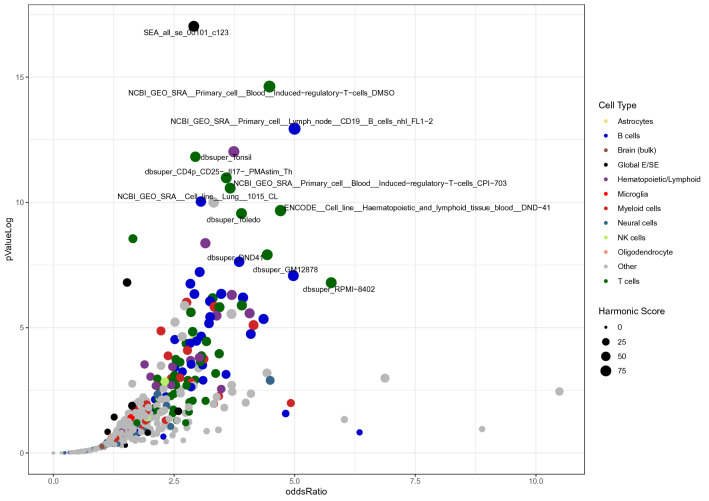
Enrichment of MS-associated SNPs in databases of regulatory elements, sorted by experiment/cell lines. X-axis shows the Odd Ratio, y-axis shows the − log (*p*Value); dot size is proportional to to the Harmonic Score (HS), a comprehensive estimation of the relevance of hits, as derived by merging and balancing the OR, *p*Value and Support ( i.e., the number of hits resulting from the colocalization analysis) of each match. Thus, prioritized hits are represented by dots that occupy the upper-right area of the chart. Dots are coloured by cell type. Labeled points have HS > 50. Labels were arbitrarily designated according to the database of origin and the cell lineage where the enrichment occurred. Supplementary Table S4displays in detail the results that generated this plot.

To consolidate this result and gain a deeper biological insight, we extended the colocalization analysis matching the ROI with a vast set of databases of regulatory DNA regions, including enhancers and super-enhancers, derived from experiments on diverse tissue types (a total of 4,697,782 DNA regions, plausibly coding for trRNA, were extracted from a wide variety of raw data sources; referenced in Additional File: Table S3). To improve interpretability of the results through ranking, we implemented a harmonic score (HS), based on the Odd Ratio, the − log (*p*-value), and the support of each match. Statistically significant results came from sets included in SEA, seDB, dbSuper and other single lists of enhancers and non-coding RNAs derived from experiments on diverse tissue types, not necessarily belonging to the immune cells lineages (Fig. 1, black dots; Additional File: Table S4). On another hand, we found a strong enrichment of MS-associated genetic variants in cell lines of hematopoietic lineage, including CD19 + and CD20 + B lymphocytes, CD4 + T helper cells, and CD14 + monocytes. This is in line with the GWAS data and the known immunopathogenesis of the disease, as well as with the fact that we consider a TT collection coming from a lymphoid line for the co-localization analysis. Moreover, among the significant hits, we found collections coming from brain resident cell, in particular microglial-specific enhancers, which is in line with recent reports on brain cell type-specific enhancer-promoter interactome activities, and the latest GWAS on MS genomic mapping^33,34^. Non-relevant tissues serving as controls (such as kidney, muscle, glands, etc.) scored low in the ranking, crowding the bottom-left corner of Fig. 1 (grey dots; see also Additional File: Table S4).

### Genetic and non-genetic factors for MS etiology converge in genomic regions plausibly coding for the transient transcriptome

Independent studies support the fact that MS GWAS intervals are enriched with DNA binding regions (DBRs) for protein ‘transducers’ mediating non-genetic factors of putative etiologic relevance in MS, such as vitamin D deficiency or EBV latent infection^17,19^. Therefore, we further inquired whether DNA regions plausibly coding for trRNA would share these features (i.e., they colocalize with such DRBs). We set up 4 new ROIs corresponding to the DBRs for VDR, activation-induced cytidine deaminase (AID), EBNA2, and Epstein Barr nuclear antigen 3 (EBNA3C), chosen among viral or host’s nuclear factors potentially associated to MS etiopathogenesis^35–37^. The DBRs for each nuclear factor were derived from recent literature (Additional File: Table S5) and matched with the GWAS-derived MS signals to confirm and expand previous results. We found statistically significant results for VDR, EBNA2, and AID for all the SNP position extensions (± 50, 100, 200 kb up- and down-stream), while for EBNA3C significant results came out at extension of ± 100 and 200 kilobases. This finding suggests that several DBRs can impact on the MS-associated DNA intervals through colocalization (Table 2).

**Table 2:** Enrichment of MS-GWAS regions (at ± 50,100,200 kb range of extension) in lists (number in brackets in the right-most column) of DNA binding sites of human and viral molecular transducers; significant results (*p* < 0.05, corresponding to a − log (*p*) > 1.301) in bold.

	± 50 KB				± 100 KB				± 200 KB			
	− log (*p*Value)	Odds ratio	Support	Harmonic score	− log (*p*Value)	Odds ratio	Support	Harmonic score	− log (*p*Value)	Odds ratio	Support	Harmonic score
EBNA2 (6880)	**10.658**	**1.790**	**158**	**45.544**	**8.616**	**1.509**	**239**	**38.327**	**15.444**	**1.542**	**421**	**41.913**
EBNA3C (3335)	0.614	1.108	55	11.765	**1.647**	**1.227**	**109**	**20.956**	**3.448**	**1.294**	**199**	**28.098**
AID (4823)	**4.963**	**1.596**	**99**	**35.793**	**3.890**	**1.374**	**153**	**30.259**	**13.924**	**1.619**	**309**	**43.308**
VDR (23,409)	**19.348**	**1.575**	**474**	**43.564**	**19.181**	**1.422**	**767**	**39.635**	**32.090**	**1.424**	**1329**	**40.872**

**Figure 2:**
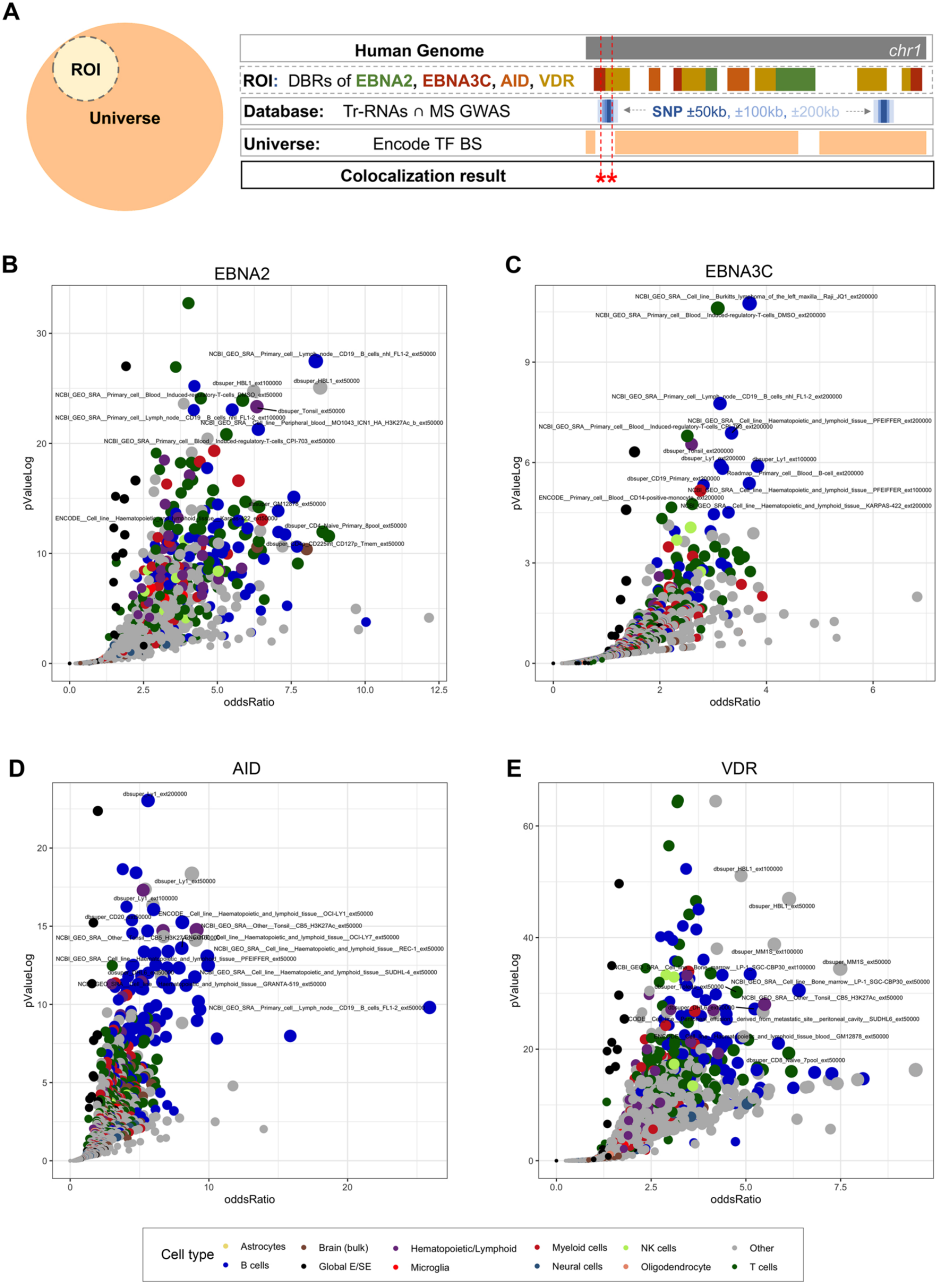
Colocalization analysis of DBRs for human and viral molecular transducers, MS-associated SNPs and DNA regulatory regions derived from databases. (**A**) Schematic representation of the colocalization analysis. (*ROI* region of interest, *DBR* DNA binding regions, *ENCODE TFBS* transcription factor binding site). The figure shows the tracks we considered for the colocalization analyses. In brief, the ROI included the DBRs of MS-related viral and human transducers and was matched with MS-associated SNPs extended by 50, 100, and 200 kilobases that colocalize with regions plausibly coding for trRNAs (Database). As a control (Universe), we took from ENCODE the entire list of transcription factors binding sites. Results were considered significant if a colocalization was found across ROI and a Databases element without occurring in the Universe as a statistically significant match*.* (**B**–**E**)*,* Colocalization results of EBNA2, EBNA3C, AID, VDR. The charts display results of all matches, i.e., with MS-associated SNPs and their extension at ± 50, 100, 200 kb. X-axis shows the Odd Ratio, y-axis shows the − log (*p*Value); dot size is proportional to to the Harmonic Score (HS). Thus, prioritized hits are represented by dots that occupy the upper-right area of the chart. Dots are coloured by cell type. Top-scoring hits in each subgroup are labeled; labels were arbitrarily designated according to the database of origin and the cell lineage where the enrichment occurred.

Building once again on the work by Michel et al.^25^, we inquired whether there was a colocalization between genomic regions containing MS-associated variants, DBRs for VDR, EBNA2, EBNA3C, AID, and DNA intervals plausibly coding for trRNA. To this end, we considered the transient transcriptome that proved to be enriched with MS-associated variants (Table 1), and we then matched the corresponding coding regions with the DBRs for the four molecular transducers. For this analysis DBRs for EBNA2 (6880 regions), EBNA3C (3835 regions), AID (4823 regions), and VDR (23,409 regions), represented the ROI, while the ENCODE database of Transcription Factors Binding Sites served as Universe (13,202,334 regions; Fig. 2a). We report the results of this analysis in Table 3, which shows the significant colocalization of DNA regions plausibly coding for trRNAs with both MS-relevant GWAS signals, and DBR of 3 out of 4 factors active at nuclear level, and potentially associated with MS. The DBR for EBNA3C did not reach statistical significance, though it showed higher values of support for short half-life transcripts.

**Table 3:** Colocalization of human and viral transducer DBRs and MS-GWAS positions (at ± 50,100,200 kb range of extension) in DNA regions coding for transient transcripts; significant results (*p* < 0.05, corresponding to a − log (*p*) > 1.301) in bold.

	± 50 KB ± 50 KB				± 100 KB				± 200 KB			
	− log (*p*Value)	Odds ratio	Support	Harmonic Score	− log (*p*Value)	Odds ratio	Support	Harmonic score	− log (*p*Value)	Odds ratio	Support	Harmonic score
**EBNA2**												
Long half-life	0.023	0.478	3	0.644	0.062	0.717	8	1.708	**1.879**	**1.531**	**33**	**24.679**
Short half-life	**6.163**	**1.920**	**69**	**43.011**	**3.241**	**1.433**	**95**	**29.496**	**8.945**	**1.610**	**189**	**40.642**
**EBNA3C**												
Long half-life	0.064	0.572	2	1.669	0.006	0.321	2	0.185	0.182	0.914	11	4.500
Short half-life	0.070	0.794	16	1.923	0.023	0.752	28	0.661	0.066	0.875	58	1.841
**AID**												
Long half-life	0.089	0.682	3	2.303	0.283	1.024	8	6.477	0.051	0.726	11	1.432
Short half-life	**1.769**	**1.465**	**37**	**23.531**	**1.346**	**1.267**	**59**	**19.367**	**3.954**	**1.442**	**119**	**31.416**
**VDR**												
Long half-life	**1.737**	**1.502**	**32**	**23.571**	0.845	1.187	45	14.646	**2.315**	**1.322**	**97**	**25.031**
Short half-life	**2.221**	**1.239**	**152**	**23.734**	**2.336**	**1.181**	**267**	**23.460**	**11.478**	**1.367**	**548**	**36.561**

The transcript half-life is considered short if < 60′ and long if ≥ 60′, respectively.

To review and confirm previous colocalizations, we considered the genomic regions resulting from the above reported match between the MS-associated GWAS intervals and the databases of regulatory DNA regions, containing enhancers and super-enhancers, plausibly enriched in trRNA-coding sequences (Fig. 2 and the online data resource). We therefore matched these DNA regions with the DBR for VDR, EBNA2, EBNA3C and AID, finding significant enrichments that allow to contextualize and prioritize genomic positions, cell/tissue identity or cell status associated to MS. Considering the harmonic score obtained from these colocalization analyses, the top hits in EBNA2, EBNA3C, and AID involved lymphoid (CD19 + B cell lines and lymphomas; T regulatory cells; tonsils) and monocyte-macrophage lineages (peripheral macrophages; dendritic cells) from experiments included in the ENCODE, dbsuper, roadmapEpigenomics databases; however, also global collections of superenhancers/enhancers and brain resident lineages appeared far from the bottom-left corner of Fig. 2 (the control datasets) (Fig. 2A–C, see also Additional File: Table S6 and the online resource). Even though immune cells prevailed also in VDR top hits, a less stringent polarization was seen, somehow reflecting the wide-spreading actions of this transducer in human biology (Fig. 2D). However, with a more stringent cutoff of Harmonic Score > 40 that selects the most significant hits (Fig. Supplement 2), a core subset of MS-relevant cell lineages, shared across all four examined transducers, became evident (Additional File: Table S7).

### A data resource for future research on transcriptional regulation in MS

**Figure 3:**
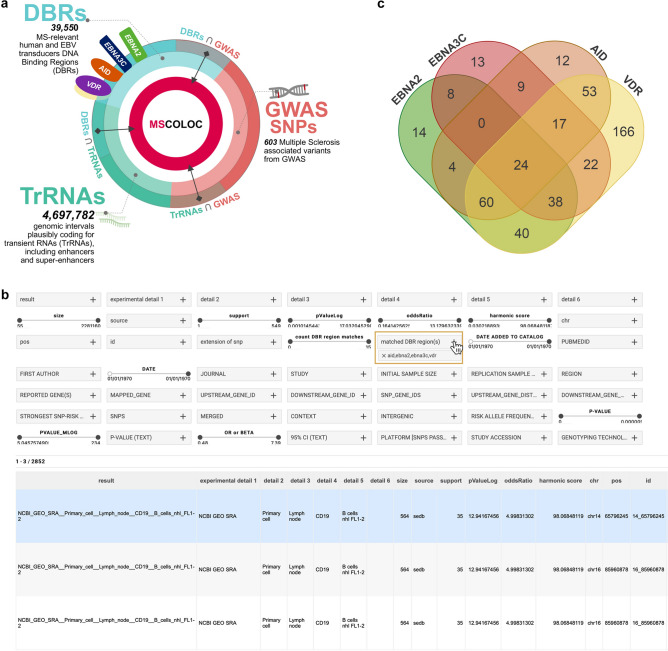
A comprehensive genomic atlas on gene-environment interactions regulating transcription in MS. (**a**) Searchable results at mscoloc.com derive from the matches of GWAS MS regions, DNA binding regions of selected genomic transducers, and more than 4 million of regions annotated as plausible transient RNAs. (**b**) The user interface includes text panels and range sliders allowing extremely personalized queries, that combine statistical significance level (including Odd Ratio, *p*Value, support, and Harmonic Score), study source, SNP or reported gene, and so on. Filtered results are shown as tables ranked by HS, that can be saved, printed or shared through URL. In the example, the cursor selects ‘*AID, EBNA2, EBNA3C, VDR’* in the ‘*matched DBR region (s)’* panel looking for MS-associated SNPs (from the ROI, Additional File: Table S1) and their extensions at ± 50, 100, 200 kb that colocalized within DNA binding regions of the molecular transducers. The top hit represents the colocalization of the DBRs, a super-enhancer region derived from experiments on CD19 + B cells included in *sedb,* and the rs8007846 MS-associated SNP on chromosome 14. (**c**) The Venn diagram shows the number of non-redundant MS-associated SNPs derived from the query: for each transducer, SNPs are considered only once if present in more than one match. Intersections show the numbers of regions colocalizing with DBRs of multiple transducers. For instance: 8 regions colocalize with both EBNA2 and EBNA3C DBRs, but not with AID nor VDR DBRs; 24 regions colocalize with all four DBRs, and could be identified as regulatory “hotspots” in MS.

A public web interface for browsing the results of our colocalization analysis is freely available at www.mscoloc.com. This is a comprehensive genomic atlas disentangling specific aspects of MS gene-environment interactions to support further research on transcriptional regulation in MS. It includes the whole list of results derived from ROI, DBRs and database matches (Fig. 3a) across all performed experiments that yielded significant results. The user can navigate across the results and perform tailored queries searching and filtering for a variety of parameters, including MS-associated variant, DBR, experimental cell type, other match details (see Fig. 3b for all available search and filter modalities). Moreover, personalized HS, *p*-value, support and Odd Ratio threshold can easily be set to screen results, that are readily displayed in tabular format. To provide an example, we select *“AID, EBNA2, EBNA3C, VDR”* in the ‘*Matched DBR region (s)*’ panel and obtain the list of MS-associated SNPs (that proved to be enriched in genomic regions plausibly coding for trRNA) targeted by all four transducers (Fig. 3b,c). Through this approach we searched for MS-associated regions shared by the DBRs analyzed, and we were able to prioritize 275 genomic regions (almost half of the MS-associated GWAS SNPs) capable of binding at least 2 molecular transducers. These regions are ‘hotspots’ of interactions between genetic and nongenetic modifier of MS risk/protection: all four proteins (VDR, AID, EBNA2, EBNA3C) proved to target 24 regions, 3 of them 115 regions, and 2 of them 136 regions. A detailed legend and more example queries may be found on the online data resource website.

**Figure 4:**
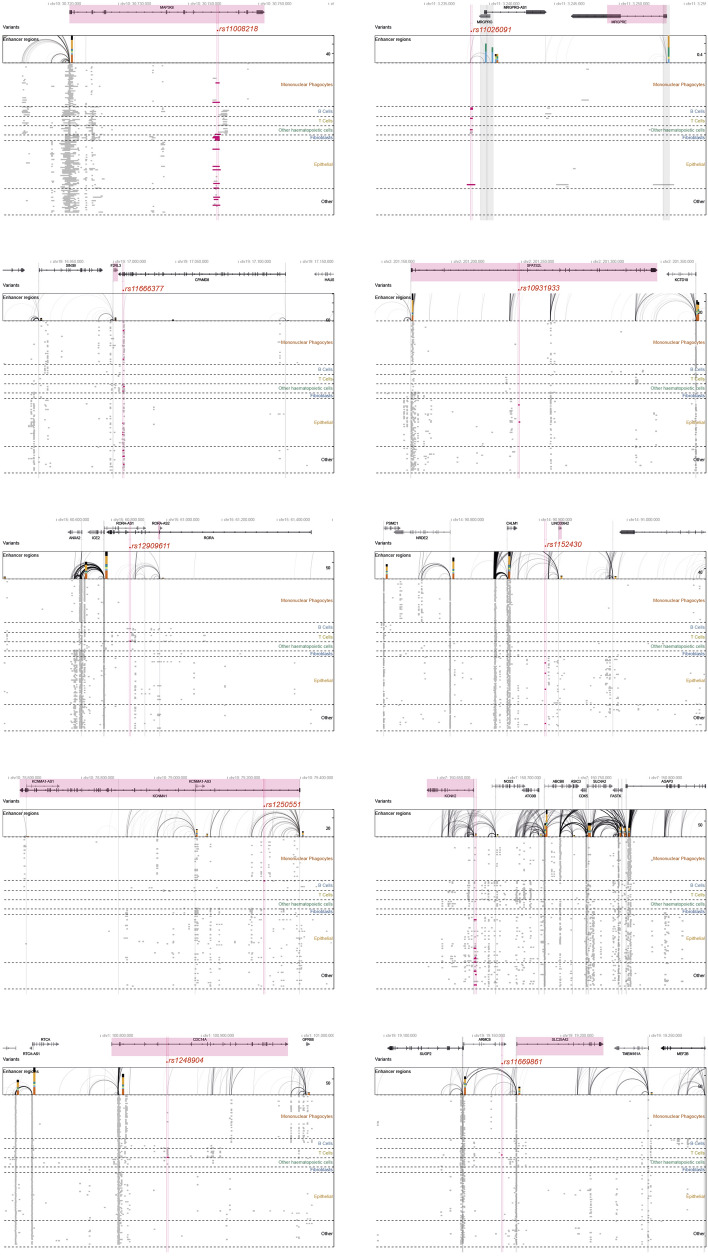
ABC gene-enhancer mapping of MS-trRNAs regulatory hotspots. The figure displays the positional mapping of selected MS-trRNAs-DBRs hotspots, their colocalization with regions encoding for regulatory elements active in specific cell types (*Mononuclear phagocytes, B cells, T cells, Other haematopoietic cells, epithelial cells, Other),* and the enhancer-gene connections that determine the ABC mapping. The gene with the highest ABC score for each hotspot is highlighted in pink.

Finally, to obtain a functional mapping of MS-TrRNA regions, we attempted to identify MS-relevant genes by integrating our results with the ‘activity-by-contact’ (ABC) model (Fulco et al., 2019; Nasser et al., 2021), which was recently developed to define cell-specific, gene-enhancers connections according to chromatin conformation and accessibility, as well as to histone acethylation-methylation status. We retrieved a total of 77 gene-enhancers pairings (Additional File: Table S8), enriched in *IL6-JAK-STAT3*, *IL-18*, *IL2RB* pathways. Among these, we focused on MS variants-trRNA colocalization hotspots targeted by all four (AID, EBNA2, EBNA3C, VDR, n = 24) or three (AID, EBNA2, VDR, n = 60; see also Fig. 3c) molecular transducers, excluding EBNA3C, as it did not reach statistical significance in previous analysis (Table 3): ABC gene-enhancers connections were found for for 10 out of 84 hotspot SNPs, corresponding to 31 genes (Table 4 and Fig. 4). As expected from the pleiotropy of enhancer activity, many MS-trRNA hotspots were linked to multiple genes differentially regulated in distinct cell types: for example, the MS-trRNA hotspot in *rs11026091* was linked to *MRGPRE* in T cells and *MRGPRG-AS1* in B cells (see also Additional File: Table S9). Results included regulators of immune cell activity (*MAP3K8, GIMAP8, TMEM176A, TMEM176B*), ion channels and solute carriers (*KCNH2, KCNMA1, SLC25A42)*, and transcriptional modulators (*ICE2, SIN3B, NWD1).*

**Table 4:** Activity-By-Contact (ABC) functional mapping of MS-trRNA hotspots bound by 4/4 MS-relevant transducers or only 3/4 (AID,EBNA2, VDR).

MS SNP-TrRNA hotspot	Transducers	Chr	Position	GWAS catalog reported gene	ABC linked gene
*rs11026091*	4/4	11	3,238,579	MRGPRE	**MRGPRG-AS1**, MRGPRG, **MRGPRE**
*rs11008218*	4/4	10	30,744,074	SVILP1	**MAP3K8**
*rs12048904*	4/4	1	100,865,980	EXTL2	GPR88, **CDC14A**, RTCA-AS1, RTCA-AS1
*rs11666377*	3/4	19	17,007,623	CPAMD8	**F2RL3**, HAUS8, MYO9B, NWD1, SIN3B
*rs11669861*	3/4	19	19,166,593	MEF2B, MEF2BNB-MEF2B	**SLC25A42**
*rs12909611*	3/4	15	60,865,204	RORA	ICE2,RORA-AS1,**RORA-AS2**
*rs1250551*	3/4	10	79,299,578	ZMIZ1	**KCNMA1**
*rs1152430*	3/4	14	90,906,615	RPS6KA5	CALM1, **LINC00642**, LOC105370619, LOC105370622
*rs11762408*	3/4	7	150,676,640	GIMAP6	ABCB8, AOC1, GIMAP8, **KCNH2**, TMEM176A, TMEM176B
*rs10931933*	3/4	2	201,247,748	CASP8	**SPATS2L**, KCTD18

Genes who scored the highest ABC score are highlighted in bold.

Moreover, in most cases, the ABC-identified genes differed from the candidate genes reported in MS GWAS, underscoring the relevance of integrative approaches to annotate statistical genomic associations.

## Discussion

Our study supports the hypothesis that investigations on the transient transcriptome may contribute to clarify how the GWAS signals affect the etiopathogenesis of MS and possibly of other complex disorders. Specifically, we show that genomic regions coding for the transient transcriptome recently described in T cells^25^, are significantly enriched for both MS-associated GWAS variants, as well as for DNA binding sites for protein ‘transducers’ of non-genetic signals, chosen among those plausibly associated to MS. The colocalization of GWAS intervals and some DNA-binding factors involved in MS etiology has already been reported^17–19^. Here we reinforce this premise and extend the result to AID, whose DBRs were not previously correlated to MS-associated genetic signals. The result is of relevance considering the role of AID in B cell biology and the high effectiveness of B cell-depleting approaches recently introduced in clinical practice to tackle the disease progression (Cencioni et al. 2021). Our colocalization analysis suggests a model in which trRNA-coding regions are hotspots of convergence between genetic ad non-genetic factors of risk/protection for MS. These hotspots are shared by two or more of the chosen transducers, indicating possible additive pathogenic effects or a multi-hits model to reach the threshold for MS development (see Fig. 3c and Additional File: Table S4). This model may reconcile previous evidences coming from ours and others’ studies on MS etiology: genetic susceptibility plausibly exerts a soft effect (with the notable exception of the major histocompatibility complex variants, that are known to directly shape the repertoire of the (auto-)immune effectors); in fact, single base changes in GWAS loci could conceivably lead to subtle changes in TT expression, and twin studies in Mediterranean areas showed a disease concordance as low as 1 out of 10 identical twin pairs (Ristori et al. 2006). A likely higher weight has the non-genetic component, that seems to be multiple and heterogenous (with the notable exception of EBV, the most recurring and convincing risk factor for MS development; Ascherio et al., 2001), and that may favor stochastic events, by prevalently acting on genome regions coding for TT.

In homeostatic conditions, it can be hypothesized that DNA sequences coding for trRNA are composed of regulatory regions where genetic variability and non-genetic signals interact to finely regulate the gene expression according to cell identity, developmental or adaptive states, and time-dependent stimuli. As a matter of fact, the sequence variability of these regions and the strict time-dependence of their transcription could be instrumental to adaptive features; however, these same features make these regions susceptible to become dysfunctional or to be the targets of pathogenic interaction. In some instances, these detrimental interactions come from outside the cell, such as in the case of EBV interference with host transcription^38,39^, and the pathogenic consequences of vitamin D deficiency; in other cases, the dysfunction develops within the cell, such as the tumorigenic activity of AID in B cells^40,41^.

To support the relationship between trRNA and transcription of regulatory DNA regions, we matched a large dataset of enhancers and super-enhancers with MS-GWAS signals and DBR for VDR, EBNA2, EBNA3C and AID. The significant enrichment in cell lines and cell status coming from the hematopoietic lineages and the CNS-specific cell subsets corroborates data coming from recent reports showing the relevance of contextualizing and prioritizing the role of MS-associated GWAS signals^33,34,42,43^). Our analysis supports the pivotal regulatory role of enhancer transcription (i.e., a main component of transient transcriptome) that was recently reported as not dispensable for gene expression at the immunoglobulin locus and for antibody class switch recombination^44^, though more research is needed to unravel such topic at a finer grain.

Reports on the dynamics of time-course data are a recent area of focus within the analysis of gene expression, specifically in immune cells. Although current studies use methods that investigate time points related to the stable transcriptome (RNA-seq performed with time spans of hours), they clearly show that gene expression dynamics may influence allele specificity, regulatory programs that seem to depend on autoimmune disease-associated loci, and different transcriptional profiles based on cell status after stimulation^45^. A recent work showed that an *IL2ra* enhancer, which harbors autoimmunity risk variants and was one of the first MS-associated loci from GWAS, has no impact on the gene level expression, but rather affects gene activation by delaying transcription in response to extracellular stimuli^46^. The importance of the timing in the gene expression control emerges also from several studies implicating enhancers and super-enhancers in the process of phase separation and formation of nuclear condensates, where the transcriptional apparatus steps-up to drive robust genic responses (Sabari et al., 2018). The overall process seems to be highly dynamic, with time spans of seconds or minutes, and hence compatible with the temporal features of the transient transcriptome, which could somehow contribute to the formation of these phase-separated condensates.

We suggest that studies on transient transcriptomes may integrate previous RNA-seq data in accounting for the interplay between genetic variability and non-genetic etiologic factors leading to MS development. Possible correlation between transient and persisting transcriptome obtained in ex-vivo and in-vivo experimental settings of neuroinflammation may help to better decipher the genomic regulatory syntax driven by non-coding DNA variants. In this context our results on ‘hotspots’, MS-associated trRNAs, and those obtained in the paper describing ABC mapping (Nasser et al., 2021) are concordant in identifying regulated additional genes, besides those resulting from current interpretations on GWAS data (Table 4 and Additional File: Table S6), thus revealing a complex scenario in cell-specific gene-enhancers interaction that supports the need of a wider approach in characterizing plausibly causal genes.

Components of a more-complex-than-anticipated regulation of gene expression could include transcriptional noise, transitory time-courses, erratic dynamics, and highly flexibility of some DNA regions, possibly oscillating between bistable states of enhancer and silencer^47^. Our analysis provides a platform for future studies on transient transcriptome, which we support by making our data resource available at www.mscoloc.com. New gene regulatory models may emerge from this approach in order to better evaluate the meaning of GWAS in complex traits and the impact of the enhancer transcription^44^, which was recently reported as an ancient and conserved, yet flexible, genomic regulatory syntax^48^.

## Methods

### Data sources

Analyses were performed in Python and R. A data freeze was applied on 3/1/2020. All GWAS data was gathered from the GWAS Catalog through its REST API^32^; about 1.5% of this data was filtered out as part of a QC process aimed at homogenizing legacy and more recent data. The MS GWAS regions were extracted from the overall GWAS Catalog data filtering by trait EFO_0003885. All Transcription Factor Binding Site regions (TFBS) were obtained from the ENCODE portal^49^. All data was organized in various databases and data pipelines as detailed below. A modular and parallel data pipeline was created to: (1) readily generate and evaluate all experiments in the paper, (2) manage and organize all data coming from various region collections (42,075 ROI regions; 4,697,782 regions plausibly coding for trRNAs; 13,309,757 Universe regions), multiple ROIs (MS GWAS, EBNA2, EBNA3C, VDR, AID, etc.), databases of vast background regions as they were populated with the data obtained from GWAS Catalog, ENCODE, and other raw data sources, (3) provide overlaps and intersection among various data elements, annotate them with the original MS GWAS loci that generated the signal, and (4) generate the overarching data resource available at www.mscoloc.com.

### ABC functional mapping

The Activity-By-Contact model was applied to map genes regulated by selected MS-trRNA colocalization hotspots. Briefly, this model identifies gene-enhancers connection taking into account chromatin accessibility (ATAC-seq and DNase-seq experiments), histone modifications (H3K27ac ChIP–seq), and chromatin conformation (Hi-C)^50^. ABC analysis was performed using the ABC pipeline outputs for 131 cell types and tissues^51^. Gene-enhancers maps were produced through https://flekschas.github.io/enhancer-gene-vis/. Pathway and process enrichment analysis of mapped genes with the highest ABC score for each coloc region was performed through Metascape^52^, using the entire human genome as background and the following ontology sources were used: GO Biological Processes, KEGG Pathway, Reactome Gene Sets, Hallmark Gene Sets, Canonical Pathways, BioCarta Gene Sets and WikiPathways.

### Statistical analysis

For SNP overlaps and region colocalization, we used LOLA^53^ and Fisher’s exact test with False Discovery Rate (Benjamini-Hochberg) to control for multiple testing. Linkage disequilibrium was considered as described in Sheffield & Bock, 2016. Resulting -log (p-value), support, and Odds Ratio (OR) were combined into a single score inspired by the harmonic mean^54^ and multi-objective optimization^55^ with the formula below, where the spacing parameter *k*_*p*_ was set to 10.0 and we consider all three contributors equally, setting therefore weights *w*_*i*_ to 1.0. Statistical significance was taken at *p* < 0.05.

$$ {Harmonic}_{{{{Score}}}} = k_{p} *\frac{{\mathop \sum \nolimits_{i}^{ } w_{i} }}{{\frac{{w_{1} }}{ - logP} + \frac{{w_{2} }}{Supp} + \frac{{w_{3} }}{OR}}} $$

For pathway analysis in Metascape, enrichment p-values were calculated based through the accumulative hypergeometric distribution, q-values were calculated using the Benjamini–Hochberg method to account for multiple testing.

## Supplementary Information

Supplementary Information 1.

Supplementary Information 2.

Supplementary Information 3.

## Data Availability

The dataset supporting the conclusions of this article is available at the website www.mscoloc.com.
