# Supplementary material for: Multiple sclerosis genetic and non-genetic factors interact through the transient transcriptome
Source: Sci Rep. 2022 May 9;12:7536. doi: 10.1038/s41598-022-11444-w (PMC9085834; doi:10.1038/s41598-022-11444-w)
Supplement: Supplementary file 1 — Supplementary Information 1. [file 41598_2022_11444_MOESM1_ESM.pdf]

Multiple Sclerosis genetic and non-genetic factors interact through the transient transcriptome

Additional Files: Index

Tables

S1. MS-associated genomic positions from GWAS catalog after QC process filtering, used as Region of Interest (ROI) for the analysis.
S2. GWAS Catalog References for diseases considered in Figure S1.
S3. Sources of DNA regions plausibly coding for trRNAs with references.
S4. Detailed results of the colocalization analysis ROI (MS) <> databases of putative TrRNAs (ref. to Figure 1).
S5. Sources of DNA Binding Regions (DBRs) of considered viral and human transducers with references
S6. Top 10 results of Colocalization analysis for each DBR.
S7. Cell types for which the colocalization analysis hits reported a harmonic score >40 in all transducers (EBNA2, EBNA3C, AID, VDR).
S8. ABC gene mapping of MS-TrRNA colocalization hits (hotspots in S9)
S9. ABC gene mapping of MS-TrRNA hotspots.

Fig. S1: Disease-associated SNPs distribution across genomic partitions and their distance relative to the transcription starting site (TSS).
Fig. S2: Harmonic Score threshold defining the top colocalization hits.
