# Supplementary material for: Multiple sclerosis genetic and non-genetic factors interact through the transient transcriptome
Source: Sci Rep. 2022 May 9;12:7536. doi: 10.1038/s41598-022-11444-w (PMC9085834; doi:10.1038/s41598-022-11444-w)
Supplement: Supplementary file 3 — Supplementary Information 3. [file 41598_2022_11444_MOESM3_ESM.pdf]

Supplementary Information: Figure Supplement S1

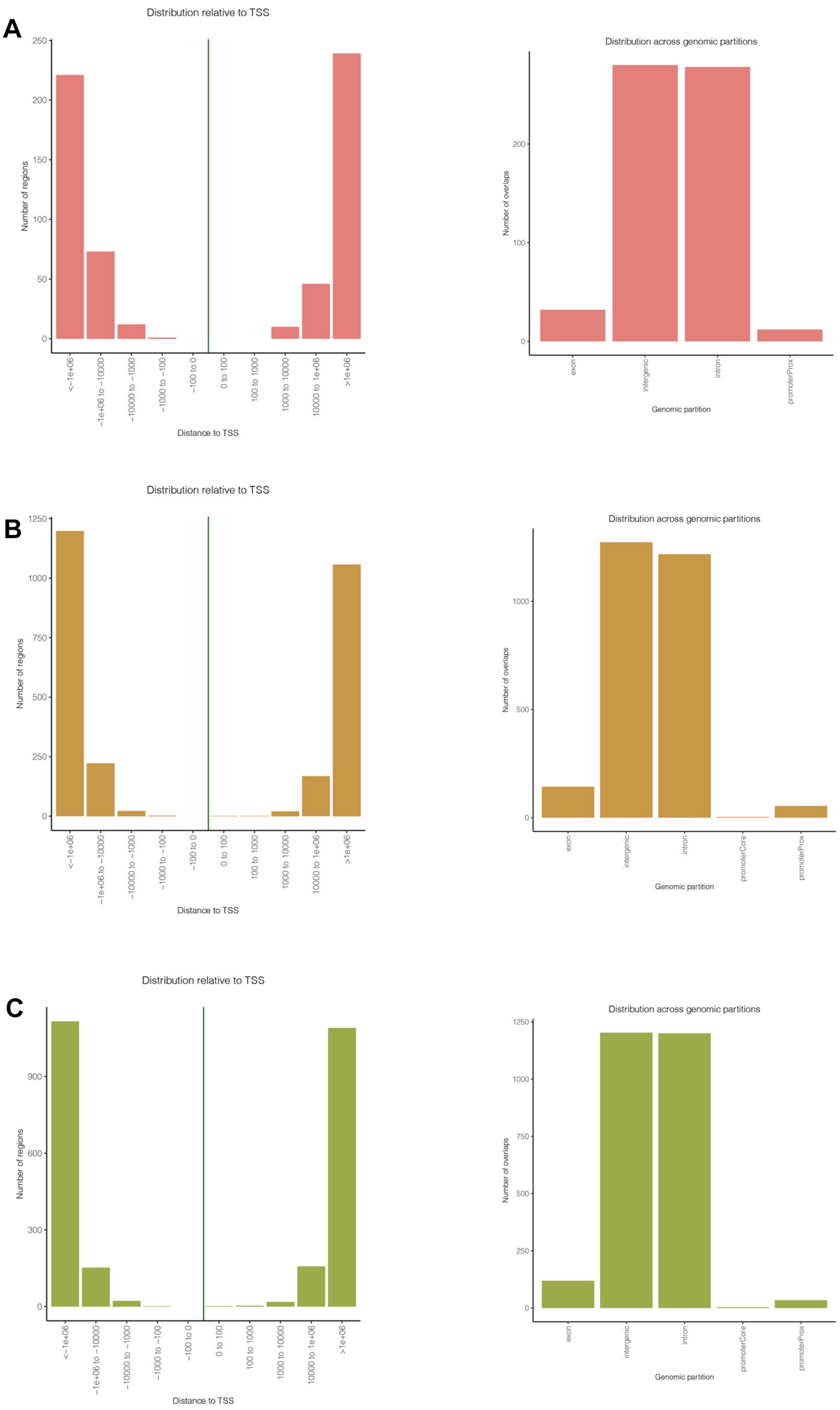

Figure S1. Disease-associated SNPs distribution across genomic partitions and their distance relative to the transcription starting site (TSS). Panel A, Multiple Sclerosis; B, Immune-mediated conditions: Multiple Sclerosis, Rheumatoid Arthritis, Systemic Lupus Erythematosus, Crohn's Disease, Ulcerative Colitis, Inflammatory Bowel Disease, Celiac Disease, Asthma, Type I Diabetes Mellitus; C, Non-immunological complex conditions: Type II Diabetes Mellitus, Aging, Obesity, Hypertension, Coronary Artery Disease, Bipolar Disorder. Supplementary Table S2 include links to these traits in the GWAS catalog.

Supplementary Information: Figure Supplement S2

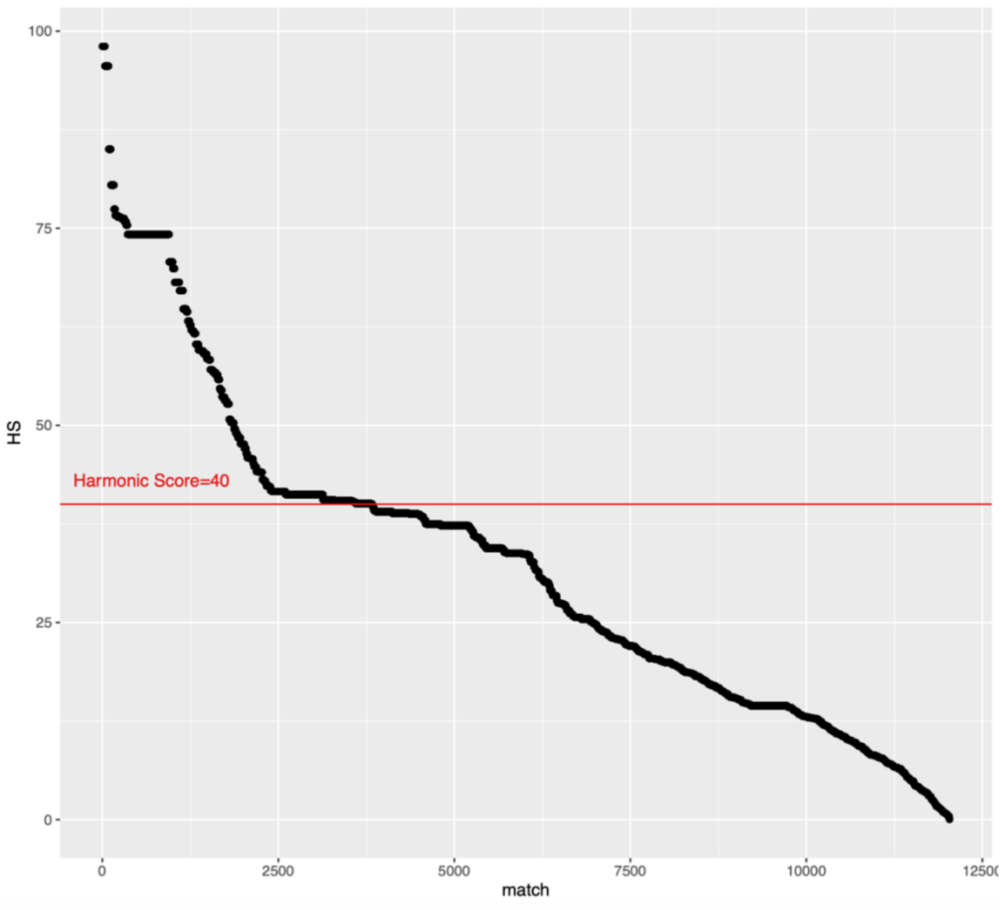

Figure S2. Harmonic Score threshold defining the top colocalization hits. The plot shows all of colocalization matches, ranked by Harmonic Score (HS). The curve inflection point, highlighted with a red line, suggests a threshold for selecting the most relevant hits (i.e., those scoring at HS>40).
